# Supplementary material for: “COVID Is Another Layer of Problematic Things”: Change, Vulnerability, and COVID-19 among University Students
Source: Int J Environ Res Public Health. 2022 Nov 30;19(23):15947. doi: 10.3390/ijerph192315947 (PMC9739650; doi:10.3390/ijerph192315947)
Supplement: Supplementary file 1 [file ijerph-19-15947-s001.zip › Qualitative Interview.EJS.pdf]

Campus COVID-19 Study: Follow-up Qualitative Interview Guide

- Tell me more about your decision to sign up for the COVID-19 antibody test. What motivated that decision? Describe any concerns you had about taking the test (probe re stigma and access to care)?
- What was the first thing you thought or did upon learning your result?
- How has the COVID-19 epidemic changed your life?
    - Daily activities? Priorities? Goals and plans? Relationships? Physical health? Mental health? Access to non-Covid-19 related health services?
- Do you know anyone who has or has had COVID-19? If yes, tell me about that person's experience with the illness.
- What kinds of worries do you have when it comes to COVID-19?
    - Self? Family? Future? (If no worries – what are your top health concerns?)
- How about your peers? What kinds of worries do they have around COVID-19?
- How do you protect yourself against getting COVID-19? What motivates you to protect yourself?
- What about your peers? How do they protect themselves (or not) from getting COVID-19?
- What do you miss most about our pre COVID-19 world?
- Has COVID-19 taught you anything about yourself?
- What can colleges do to better understand students' needs around COVID-19? Better deliver prevention and safety messages around COVID-19?

For those who tested positive for COVID-19 antibodies as part of the Campus Study in Fall 2020, additional/slightly altered questions were asked:

- What was it like to hear your result? How would you describe your reaction? Who did you tell – or not tell? I'd like to hear more about those decisions…
- What concerns did you or do have about the way others will treat you because of COVID-19?

Before learning about the results of your antibody test –

- How had the COVID-19 epidemic changed your life?
    - Daily activities? Priorities? Goals and plans? Relationships? Physical health? Mental health? Access to non-Covid-19 related health services?
- What kinds of worries did you have about COVID-19?
    - Self? Family? Future?
- What kinds of things were you doing to protect yourself from COVID-19?

After learning about your antibody test –

- What changed about your concerns? The way you protect yourself? Your relationships? Your activities?
- Do you know anyone who has or has had COVID-19? If yes, tell me about that person's experience with the illness.
- I also want to know more about your peers – how much do they worry about COVID-19?
- How do they protect themselves (or not) from getting COVID-19?
- What do you miss most about our pre COVID-19 world?
- Has COVID-19 taught you anything about yourself?
- What can colleges do to better understand students' needs around COVID-19? Better deliver prevention and safety messages around COVID-19?
