# Supplementary material for: “COVID Is Another Layer of Problematic Things”: Change, Vulnerability, and COVID-19 among University Students
Source: Int J Environ Res Public Health. 2022 Nov 30;19(23):15947. doi: 10.3390/ijerph192315947 (PMC9739650; doi:10.3390/ijerph192315947)
Supplement: Supplementary file 1 [file ijerph-19-15947-s001.zip › Table S1.pdf]

Table S1: Participant demographic information

**Gender***	**N**	**%**
Female	20	60.6
Male	12	36.4
Non-Binary	1	3.0
**Race/Ethnicity**		
White	16	48.5
Non-white**	17	51.5

*Include 10 students who identified as LGBTQ

**Non-white include students who identified as Hispanic, Black or Mixed-race.
