# Supplementary material for: “COVID Is Another Layer of Problematic Things”: Change, Vulnerability, and COVID-19 among University Students
Source: Int J Environ Res Public Health. 2022 Nov 30;19(23):15947. doi: 10.3390/ijerph192315947 (PMC9739650; doi:10.3390/ijerph192315947)
Supplement: Supplementary file 1 [file ijerph-19-15947-s001.zip › Table S2 supplementary quotes.EJS.pdf]

Table S2. Supplementary quotes from participants

Theme	Participant race/ethnicity & gender	Quote
**Tested mental health**	White male	Emotionally, I was just exhausted, and just so stressed with how everything changed. And I think it has been difficult kind of in different waves, not getting to see people, loss of that social interaction has definitely been hard emotionally.
	White female	I would say definitely, a lot more anxiety related to schoolwork, because I couldn't go in and get extra help if I needed it. And also, the format of tests being online really did stress me out a lot.
	White female	So I had to be in quarantine during Christmas and stuff like that. That put me in a very hard mental state. The fact that I wasn’t able to buy presents for anybody, because I couldn't go shopping, and stuff like that. That was difficult.
**Sensemaking: changing perspectives**	White male	Because of COVID, I’m seeing a marked decrease in hirings from universities, thinking kind of long term. I go on the job market this coming summer and universities aren't really bouncing back; the impact of the pandemic on universities is ongoing. And so what that looks like for job opportunities is worrisome.
	White female	I think one of the biggest things that I’m worried about is just that, like, it’s not going to stop (*referring to the COVID-19*), essentially, that it’s going to keep going, because it feels like, every time we get the numbers down, they start to go back up. Or the people go, “Oh, the numbers are down, so let's reopen stuff, or let’s start doing things again.” No. The numbers are down because we're not doing things. I guess that's one of the big things I’ve noticed. And then, just the general state of the world, too. This whole pandemic has given, I feel like, everybody a lot of time to analyze how the world works, and what's going on in it, and all of that. I feel like one of the biggest frustrations

		I have is that it won't change anything, and that people will just kind of go back to normal, and act as if we should just pretend like we haven't learned any lessons through all of this, and that there hasn't been kind of a point, essentially, and that there are things that we could have done to prevent this and could prevent it in the future, and stuff like that.
	White female	So, I think that's (*referring to uncertainties about the future*) like my biggest fear. How long is this going to go on? Are we going to have to wear masks forever now? Just like simple things like going to the grocery store. I feel on edge sometimes now, you know? It's like, how long is that going to go on?
**Adapting to the pandemic's impact**	Non-white female	I have had more time to focus on my personal goals throughout the pandemic because there is not a whole lot to do. In Illinois we had a major lock down from March to May. There was nothing else to do but focus on yourself and what was going on in your house. I was able to focus more on healthy eating and mental wellness. I was able to go to therapy and figure out all of that. A lot of people have gone through a lot of mental difficulties being isolated and have anxiety. I was able to reverse that and spend a lot of time on my mental health and well-being.
	White female	I think it's pretty different. The year before COVID-19, I started going to the gym, and I got really into yoga and, like, ballet, at The Rec center here. So I was, like, going multiple times a week. I was like, I'm so cool, I'm going to the gym. I loved it, and it made me feel really good. But then COVID-19 happened, and they shut it down. They offered home classes, but it wasn't the same. So, I started baking a lot, and, like, I made bread like everyone else, and I still make bread. So I guess I embraced cooking a lot more, and I made, like, pizza from scratch. I

		made a quarantine cookbook that I still use. I guess that's changed how I cook.
	Non-white female	It taught me that I am more resilient than I thought. I went through a stage of depression and anxiety when it started as I had stated. The pandemic however taught me how to think of things in different ways. I learnt how to make new friends, learn how to study differently, learn how to interact with my family differently. Being stuck in a house with them for three months nearly drove me insane but I had to figure it out. I feel like I am very resilient because of that.
